# Reduced Cardiac Vagal Modulation Impacts on Cognitive Performance in Chronic Fatigue Syndrome

**DOI:** 10.1371/journal.pone.0049518

**Published:** 2012-11-14

**Authors:** Alison Beaumont, Alexander R. Burton, Jim Lemon, Barbara K. Bennett, Andrew Lloyd, Uté Vollmer-Conna

**Affiliations:** 1 School of Psychiatry, University of New South Wales, Sydney, New South Wales, Australia; 2 School of Medical Sciences, University of New South Wales, Sydney, New South Wales, Australia; 3 Department of Medical Oncology, Prince of Wales Hospital, Sydney, New South Wales, Australia; 4 Inflammation and Infection Research Centre, School of Medical Sciences, University of New South Wales, Sydney, New South Wales, Australia; Federal University of Rio de Janeiro, Brazil

## Abstract

**Background:**

Cognitive difficulties and autonomic dysfunction have been reported separately in patients with chronic fatigue syndrome (CFS). A role for heart rate variability (HRV) in cognitive flexibility has been demonstrated in healthy individuals, but this relationship has not as yet been examined in CFS. The objective of this study was to examine the relationship between HRV and cognitive performance in patients with CFS.

**Methods:**

Participants were 30 patients with CFS and 40 healthy controls; the groups were matched for age, sex, education, body mass index, and hours of moderate exercise/week. Questionnaires were used to obtain relevant medical and demographic information, and assess current symptoms and functional impairment. Electrocardiograms, perceived fatigue/effort and performance data were recorded during cognitive tasks. Between–group differences in autonomic reactivity and associations with cognitive performance were analysed.

**Results:**

Patients with CFS showed no deficits in performance accuracy**,** but were significantly slower than healthy controls. CFS was further characterized by low and unresponsive HRV; greater heart rate (HR) reactivity and prolonged HR-recovery after cognitive challenge. Fatigue levels, perceived effort and distress did not affect cognitive performance. HRV was consistently associated with performance indices and significantly predicted variance in cognitive outcomes.

**Conclusions:**

These findings reveal for the first time an association between reduced cardiac vagal tone and cognitive impairment in CFS and confirm previous reports of diminished vagal activity.

## Introduction

Chronic fatigue syndrome (CFS) is a debilitating disorder characterised by medically-unexplained, disabling fatigue and constitutional and neuropsychiatric symptoms of at least 6 months duration [1]. The pathophysiological mechanisms underlying the production and maintenance of symptoms in CFS remain unknown, despite concentrated research efforts [2].

In CFS self-reports of cognitive difficulties are prominent [3], [4] however the search for a specific neurocognitive deficit in this disorder has not been successful. Typically, formal evaluation of cognitive impairment in CFS has revealed subtle deficits across a number of domains, which are best explained in terms of a generalised impairment in concentration, attention, or information processing speed. Such impairment would broadly affect performance in general, and account for the most consistently reported deficits in working memory and slowed response speed [3]–[5]. The cognitive deficits associated with CFS also bear similarities to those reported in patients with major depressive disorder [5]–[7]. Although mood disorder is exclusionary, patients with a diagnosis of CFS typically have high levels of psychological distress and disturbed mood [1]. It is therefore possible that depressed or anxious mood contributes to the cognitive complaints in CFS. However, attempts to relate cognitive difficulties in CFS to either subjective fatigue or disturbed mood have so far produced inconsistent findings [3], [5]–[7].

There is growing awareness that imbalance of the autonomic nervous system (ANS) can critically impact on the severity and outcome of a large spectrum of diseases [8]. The ANS has evolved to favour sympathetic dominance to enable adaptive survival ‘fight/flight’ responses in times of perceived danger. At such times, the prefrontal cortex becomes hypoactive, reducing vagal (parasympathetic) outflow and facilitating dynamic sympathetically driven responses to the threat. Chronic stress may cause prolonged hypoactivity in the prefrontal cortex leading to dominance of sympatho-excitatory circuits and loss of vagal control. This engenders a hyper-vigilant, defensive physiological state lacking dynamic flexibility that has been linked to increased morbidity and all-cause mortality [8]. As the heart is one of many organs that receive innervation by both branches of the ANS, a number of cardiac measures including heart rate (HR), HR variability (HRV) and time-to-recovery of resting HR after exposure to a stressor, provide valid indices of centrally-mediated vagal inhibition of sympatho-excitatory circuits [8]–[10].

The precise nature and extent of the involvement of the ANS in CFS is not yet determined, however, increasing evidence suggests chronic sympathetic hyper-arousal [increased resting HR and reduced HRV] in patients with CFS [11], [12] that persists even during sleep [13], [14]. While not explored in CFS, several studies have shown significant associations between reduced cardiac vagal activity and cognitive performance indices including reaction times and mental flexibility, suggesting that vagally-mediated HRV is associated with efficient attentional regulation and response flexibility [15]–[17]. Using a series of standardized cognitive tasks, the aim of the present study was to explore the relationship between current symptoms, cardiac vagal tone and self-paced cognitive performance in CFS.

## Methods

### Participants

Thirty patients with CFS were recruited from a tertiary referral clinic associated with the University of New South Wales in Sydney, Australia, which provides a graded-activity oriented cognitive-behavioural therapy program. All patients had been diagnosed according to the international diagnostic criteria for CFS [1]. Forty control subjects were recruited by advertisement from the local community. The groups were matched for age, sex, body mass index (BMI), hours of weekly exercise, and education.

Exclusion criteria for the study were: pregnancy, primary sleep disorder, endocrine, neurological, autoimmune or cardiovascular disease, and any major depressive, psychotic or substance abuse disorder. Medications known to affect autonomic functioning including beta-blockers, benzodiazepines, corticosteroids, and any other centrally-active drugs were also exclusionary.

### Ethical Approval

The Human Research Ethics Committee of the University of NSW approved this research (HREC Approval No: 08085). The study was conducted in accordance with the principles expressed in the Declaration of Helsinki. All participants gave written informed consent before taking part.

### Procedure

Testing was carried out at a comfortable ambient temperature (23±3°C). Participants were asked to abstain from caffeine, alcohol and exercise for 12 hours prior to testing. Participants were connected to physiological sensors and sat in a semi-reclined relaxed position for 5 minutes during baseline recordings. Participants then rated their current sensations of ‘physical’ and ‘mental’ fatigue and perceived energy levels on a series of 1–10 Likert scales (1  =  none at all, to 10  =  absolutely the most possible) before and after cognitive testing. Presentation of the first two tests was counter-balanced to control for possible order or carry-over effects on performance, including fatigue and learning. However, the Stroop task was always presented last as previous findings by our group found that this cognitive task generally provided greater autonomic activation than the other cognitive tasks used in this study. At the completion of the Stroop task, participants were asked to relax until HR had returned to baseline.

### Questionnaires

Questionnaires were used to obtain medical history and demographic information and to assess health behaviours, symptoms, and functional impairment. Education was assessed as years of education completed. The number of hours/week of exercise sufficient to cause a noticeable increase in HR and breathing (e.g. brisk walking) was recorded.

The 34-item Somatic and Psychological Health Report (SPHERE) [18] assessed a wide range of physical and psychological symptoms; with an empirically-derived, validated subscale (the SOMA) identifying the key clinical features of prolonged fatigue states. The Kessler 10 (K10) produced a global measure of “psychological distress” based on questions about the level of anxiety and depressive symptoms [19]. The Brief Disability Questionnaire (BDQ) [20] assessed functional impairment, specifically the ‘days in bed’ and ‘days out of role’ were quantified days over the past month during which the respondent stayed in bed or was unable to carry out usual daily activities due to illness. The Pittsburgh Sleep Quality Index (PSQI) [21] recorded the quality and pattern of sleep. The short-form McGill Pain Questionnaire provided information on the sensory, affective and evaluative dimensions of pain experiences [22].

### Cognitive Performance Tasks

Computerised versions of three standard cognitive tests, the Digit Symbol test, Spatial Working Memory task and the Stroop-Colour-Word test were used [5], [12], [23]. These tests permit assessment of cognitive functioning across the modalities previously found to be affected by CFS (including sustained attention, working memory, and response flexibility) and produce indices of response speed, as well as performance accuracy. Additionally these modalities have been found to be affected by variation in HRV and thus the chosen tests will permit an evaluation of an association between typical cognitive difficulties in CFS and altered HRV. To obtain an accurate assessment of response speed, all tests were self-paced, requiring a response before the next trial appeared on the screen. In the digit symbol test, the digits 0 to 9 were presented each with a different symbol beneath it. One of the symbols from the set was displayed and participants were required to key in the corresponding digit. As soon as an answer was keyed in another symbol appeared. Participants were asked to complete as many of these trials as possible within a 3 minute period. The total number of answers, percentage correct and average reaction time (RT) was recorded.

The spatial working memory task consisted of a 9 square grid in which 3 squares flashed in sequence. The participant was asked to recreate this sequence by mouse-clicking the squares in the same order as the original sequence. If correct, the sequence re-appeared, extended by one additional square. The sequence continued to grow in length until the participant could no longer retrace it from memory and made an error. Following this, a new 3 square sequence began immediately. This test consisted of 6 such sequences. The maximum path length, average error position and average reaction time were recorded.

The Stroop Colour Word test presented a randomised sequence of tasks consisting of an upper case word (either NAME or COLOUR) and a lower case word red, green or blue coloured either, red, green or blue. If the upper word was ‘*NAME*’ the participant had to press the button corresponding to the *name of the word* below it. If the word ‘*COLOUR*’ appeared, the subject had to press the button corresponding to the *colour of the text* below. Participants were asked to answer as many items as possible within a 5 minute period. The percentage correct and average reaction times were recorded.

### Physiological Measures and Data Preparation

HR was monitored via standard three-lead ECG chest electrodes and respiration was monitored via a strain gauge transducer (Pneumotrace, UFI, USA) around the chest. All sensors were connected to an ML880 16 channel Powerlab using LabChart 7 software for recording and analysis (ADInstruments, Bella Vista, Australia). Physiological measures were recorded for the duration of the testing process.

At the completion of testing, the raw data was analysed in LabChart 7. HR and respiratory rate were calculated from raw ECG and respiratory recordings. Mean HR and respiratory rate were sampled in 1 minute blocks throughout the baseline, testing and recovery periods.

The root mean square of successive differences of R-R intervals (rMSSD) – a time domain measure of HRV was calculated using the HRV module (ADInstruments, Bella Vista, Australia). This measure has been shown to be an accurate index of vagal/respiratory sinus arrthymia modulation of heart rate [24]. HRV analysis was performed from the recorded ECG for a stable 3 minute period for baseline, digit symbol and Stroop recordings, and a stable 2 minute period for the spatial working memory and recovery periods.

It is well documented that following a stressor, the vagus plays a major role in restoring HR to baseline values [9], [10]. The moment of HR recovery was defined by the point at which instantaneous HR [calculated in beats per minute (bpm)] had returned to baseline values for at least 5 seconds.

### Statistical Analysis

Analyses were performed using PASW Statistics for Windows version 18 (SPSS Inc., Chicago, IL, USA). Normality of variables was ascertained by graphical methods (normal Q-Q plots). We used t-tests for simple group comparisons of participant characteristics (age, level of education, activity levels, BMI, current symptoms and functional disability); and for analysis of between-group differences of baseline values of physiological parameters and in rated fatigue and energy levels; the Chi-square test was used to assess independence of categorical data such as sex. General linear model (GLM) repeated measures ANOVA served to assess differences in repeated measures, between-group differences, and interactions between group and repeated effects for HR, HRV, as well as changes in rated fatigue, energy and effort in relation to performing the cognitive tasks. Cohen’s *d* or partial eta squared (*η_p_^2^*) were calculated to indicate size of effect. Kaplan-Meier survival analysis was used to assess differences in time-to-recovery to resting HR after the cognitive stressor. Correlational (Pearson) analyses tested bivariate associations between current symptoms or HRV and cognitive performance parameters. To further evaluate the relative importance of HRV and other variables with documented potential to affect cognition (i.e. current severity of fatigue and other somatic symptoms, and psychological distress) to outcomes in cognitive performance multiple regression modelling using the enter method was used.

## Results

### Participant Characteristics

There were no between-group differences in demographic characteristics, including age [CFS: Mean (M) = 36, standard deviation (SD) = 11.8 years versus Controls: M = 34.6, SD = 12.1; t(68) = 0.54, p = 0.59], sex ratio [Female:Male (n), CFS: 20∶12 vs Controls: 24∶16; χ^2^ (1) = 0.08 p = 0.81], hours of moderate intensity exercise [CFS: M = 3.5, SD = 5 vs Controls: M = 4.2, SD = 2.6; t(68) = 0.76 p = 0.45] or education [CFS: 67% >12 years education vs Controls: 79%>12 years; χ^2^ (1) = 1.6, p = 0.27]. There was a trend towards higher BMI in the control participants [CFS: M = 23.8, SD = 3.7 vs Controls: M = 25.4, SD = 3.0, t(68) = 1.83, p = 0.06].

The clinical characteristics of the participants are shown in Table 1. As expected, patients with CFS reported significantly higher fatigue related symptoms (SOMA), as well as common symptoms of psychological distress (K10). Compared to healthy subjects, patients with CFS were more likely to report functional impairment in performing basic daily activities (BDQ), and reported significantly more days over the past month during which they could not fully fulfil their normal roles, or remained in bed due to their illness. Patients with CFS additionally reported significantly poorer sleep quality (PSQI) and greater overall pain intensity (McGill).

**Table 1:** Clinical characteristics patients with CFS and of healthy participants.

	CFS (n = 30)	Healthy (n = 40)	*t*	*p*-value	Effect size *d*
**SOMA** *(physical symptoms)*	7.75 (3.85)	1.36 (1.93)	8.10	**<0.001**	2.10
**K10** *(psychological distress)*	22.66 (7.78)	14.31 (4.39)	5.20	**<0.001**	1.33
**BDQ** *(functional impairment)*	15.14 (3.63)	2.31 (4.69)	12.25	**<0.001**	3.06
Days out of role	17.28 (11.74)	1.69 (5.14)	6.69	**<0.001**	1.72
Days in bed	2.5 (5.68)	0.46 (1.30)	1.86	**0.030**	0.49
**PSQI** *(sleep quality)*	7.42 (4.13)	4.45 (2.63)	3.25	**0.002**	0.86
**McGill** *(pain)*	11.83 (11.45)	2.53 (3.37)	4.24	**<0.001**	1.10

Values are group means and (standard deviations). CFS  =  chronic fatigue syndrome; SOMA  =  somatic subscale of the SPHERE (somatic and psychological health report); K10 =  Kessler 10; BDQ  =  brief disability questionnaire; PSQI  =  Pittsburgh Sleep Quality Index; McGill  =  Short form McGill Pain Questionnaire.

### Cognitive Performance

Patients with CFS showed significantly slower reaction times throughout [M = 1938 ms, SD = 641] when compared to controls [M = 1681 ms, SD = 343; t(68) = 2.14, p = 0.036; *d* = 0.5]. However, there were no significant differences (all p>0.05) in performance accuracy assessed as percentage correct responses in the digit symbol test [CFS: M = 97.8%, SD = 2.2; Controls: M = 97.8%, SD = 2.8; *d* = 0], or the Stroop test [CFS: M = 95.5%, SD = 4.0; Controls: M = 96.8%, SD = 3.1; *d* = 0.33]. Additionally, there were no differences in spatial working memory performance as maximum path length [CFS: M = 6 squares, SD = 2; Controls: M = 7 squares, SD = 2; *d* = 0.5] or error position [CFS: M = 4^th^ square, SD = 2; Controls: M = 5^th^ square, SD = 2; *d* = 0.5].

### Subjective Ratings of Fatigue, Energy and Effort at Baseline and after Cognitive Testing

Patients with CFS reported significantly higher fatigue levels and lower energy levels at both the baseline [all t(68)>3.17, p<0.002; *d* >0.77] and post-test assessments [all t(68)>4.31, p<0.001; *d* >1.04]. The perceived effort required to complete the cognitive tests was significantly higher in patients with CFS [M = 6.2, SD = 1.5] compared to control subjects [M = 4.9, SD = 1.2; t(68) = 3.97, p<0.001; *d* = 0.96]. There were no significant between-group differences in the changes in energy and fatigue levels as a consequence of performing the tasks. No significant correlations were found between cognitive performance and any of the subjective ratings of energy, fatigue or effort, or the self-rated severity of other symptoms (all p>0.05).

### Heart Rate at Baseline and during Testing

**Figure 1:**
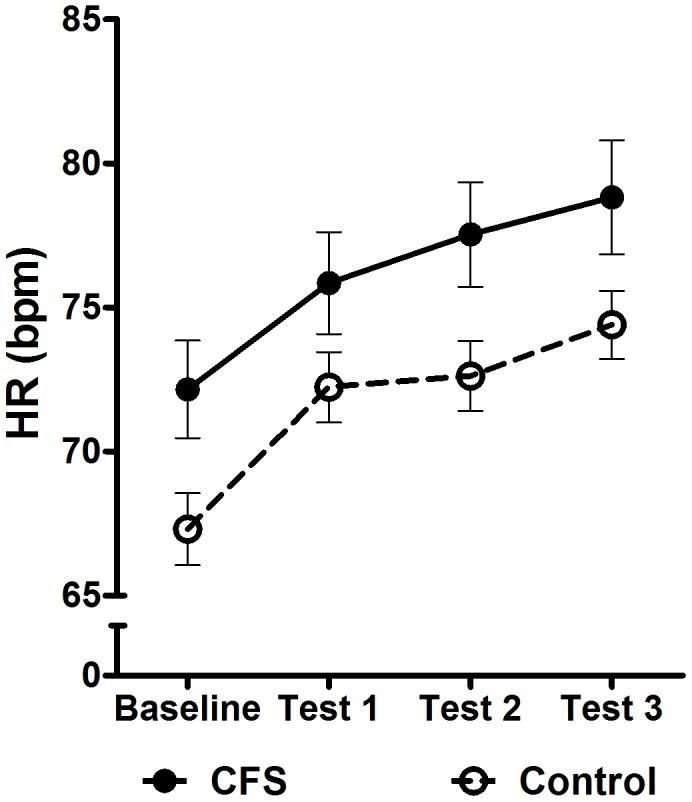
The average change in HR (as bpm) from resting baseline in response to cognitive task performance in patients with CFS and healthy control participants. Error bars represent the standard error of the mean (SEM). HR  =  heart rate; bpm  =  beats per minute; CFS  =  chronic fatigue syndrome.

There was a significant between-group difference in the average baseline HR of patients with CFS [M = 72 bpm, SD = 9] compared to controls [M = 67 bpm, SD = 8; t(68) = 2.41, p = 0.02; *d* = .58], indicating a higher resting HR in CFS. Analysis of the change in average HR over whole testing period (Figure 1) revealed a significant difference in overall HR, with patients showing higher HR [CFS, M = 76, SD = 10] compared to control participants [M = 72, SD = 7; F(1,68) = 4.83, p = 0.03; *η_p_^2^ = 0.07*]. There was also a significant group x cubic trend interaction [F(1,68) = 4.16, p = 0.04; *η_p_^2^* = 0.06]. Figure 1 reveals that the HR of control participants increased initially, then stabilized during the less demanding first and second tests, before rising again in the more demanding Stroop task. In contrast, the HR of patients with CFS continued to rise throughout the entire testing process.

### Differences in HR Recovery Times

**Figure 2:**
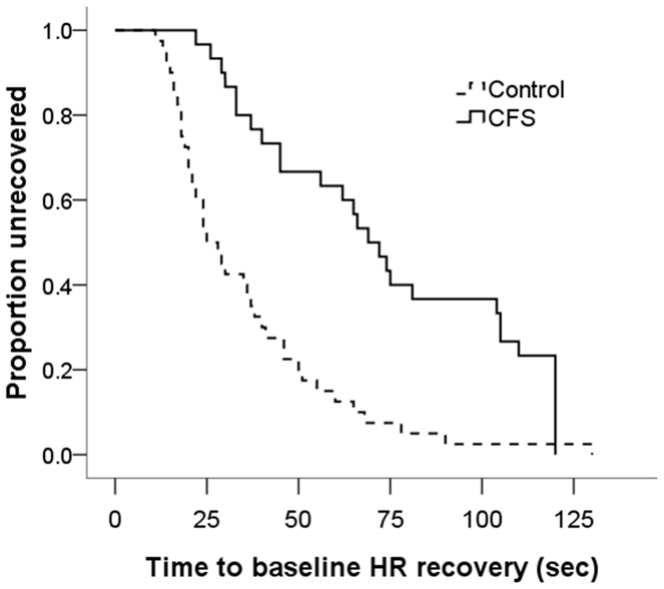
Proportion of participants who had returned to baseline HR values during the two minute recovery period. HR  =  heart rate.

Time-to-event analysis (Kaplan Meier) revealed a highly delayed average time-to-recovery of HR to baseline levels for patients with CFS [M = 74s, SD = 6.4 versus M = 36s, SD = 3.8; χ^2^ (1) = 22.04, p<0.001; Figure 2]. Compared to healthy participants, patients with CFS were 10.5 times less likely (95% CI 3.2–34.4) to achieve resting HR within the first minute of the recovery period.

### HRV at Baseline and during Testing

**Figure 3:**
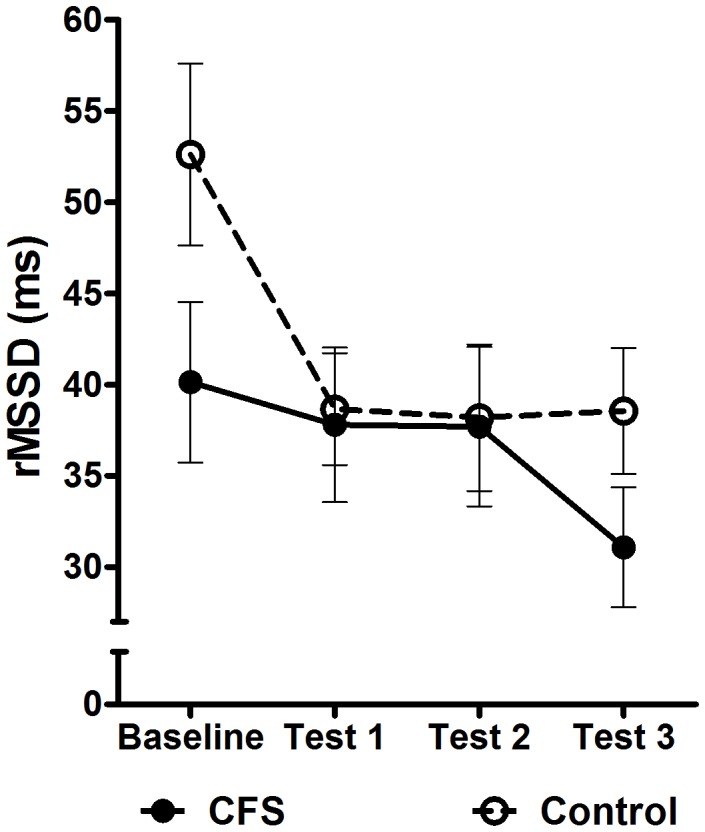
The average change in HRV (as rMSSD) from resting baseline in response to cognitive task performance in patients with CFS and healthy control participants. Error bars represent the standard error of the mean (SEM). HRV  =  heat rate variability; rMSSD  =  root mean square of successive differences of R-R intervals; CFS  =  chronic fatigue syndrome.

During baseline, there was a trend for lower HRV (as rMSSD in ms) in the CFS group [CFS: M = 40.4, SD = 22.6] compared to control participants [M = 52.6, SD = 30.0; t(68) = 1.84, p = 0.07; *d* = 0.45]. Engagement in the cognitive tasks resulted in a reduction in rMSSD in both groups. Assessment of the change in rMSSD over the entire testing period revealed a significant group x quadratic trend interaction [F(1,68) = 4.56, p = 0.04; *η_p_^2^ = *0.07]. Inspection of Figure 3 shows that the rMSSD of control participants initially dropped sharply and then stabilised for the remainder of the tests, whilst patients with CFS showed a delayed response in rMSSD, but continued to decline throughout the testing process.

Although patients with CFS had lower rMSSD overall [F(1,68) = 4.6, p = 0.04; *η_p_^2^ = *0.06], the initial response to the stressor was greater in control subjects then in patients with CFS [Controls: M = 13.6, SD = 28.0; CFS; M = 1.2, SD = 21.6; t(68) = 1.96, p = 0.05; *d* = 0.48].

There were no significant differences in the resting mean respiratory rates of the two groups [CFS: M = 14 breaths per minute, SD = 3.4; Controls: M = 12 breaths per minute, SD = 3.4; t (68) = 1.5, p = 0.13; *d* = 0.38].

### Relationship Between HRV, Symptoms and Objective Performance Parameters

To explore the hypothesis of a contribution of a loss of vagal tone to cognitive dysfunction in CFS, correlational analyses of individual differences were performed. No significant correlations were found between any of the cognitive performance indices and somatic (SOMA scores) and psychological distress (K10 scores) symptom rating, functional impairment (BDQ scores) or the subjective ratings of energy, fatigue or effort (all p>0.05). However, highly significant relationships were revealed between baseline rMSSD and performance speed, as well as indices of performance accuracy (see Table 2).

**Table 2:** Correlations between baseline rMSSD (ms) values and cognitive performance outcomes.

Cognitive outcome	*r*	*p-value*
**Digit symbol test**		
Number correct	0.283	0.020*
Reaction time	−0.262	0.032*
**Spatial working memory**		
Maximum path length	0.360	0.003*
Error position	0.305	0.012*
**Stroop task**		
Discordant RT	−0.265	0.032*
Concordant RT	−0.295	0.016*
Total RT	−0.290	0.018*

The correlations coefficients (*r*) are from Pearson correlations; rMSSD  =  root mean square of successive differences of R-R intervals; RT  =  reaction time.

To further evaluate the relative importance of HRV in cognitive performance, multiple regression modelling was performed using the condition (CFS or control), baseline HR (a covariate of HRV) and HRV, baseline reported mental and physical fatigue levels, fatigue related somatic symptoms (SOMA scores), and reported levels of psychological distress (K10 scores) as predictors of cognitive performance outcomes (total response time, and spatial working memory performance). Both models were significant (p = 0.026 and p = 0.029 respectively) and predicted approximately 25% (R^2^) of the variance in the outcome variable. Collinearity statistics were very acceptable. The mean variance inflation factor was 2.1 (range:1.2–3.1); and mean tolerance values were 0.54 (range: 0.31–0.83). HRV at baseline was the only significant predictor of overall performance speed (β = −0.38, p = 0.004) and maximum path length in the spatial working memory task (β = 0.45, p = 0.001). Thus, 1 SD decrease in resting rMSSD levels was linked to a 0.38 SD increase in reaction time; and a 0.45 SD decrease in spatial working memory performance.

## Discussion

This study found a significant reduction in cognitive performance speed in patients with CFS compared with healthy controls. The reduced cognitive performance seen in CFS was not associated with somatic symptoms, psychological distress, functional impairment, and subjective ratings of energy, fatigue or effort. It did, however, significantly correlate with baseline levels of HRV. The results additionally reveal a number of novel insights regarding autonomic activation at rest and during challenging tasks in patients with CFS, and provide the first results implicating reduced vagal activity in cognitive impairment in this condition. More broadly, our findings add to the growing body of evidence linking autonomic dysfunction to the symptomatology in this poorly understood disorder [11]–[14], [25].

### Cognitive Performance and Vagal Modulation

Although reports on the extent of neuropsychological dysfunction in CFS vary considerably, our finding of slowed response speed concurs with the most consistent outcomes in the literature and suggests a generalized difficulty to concentrate and focus attention. Between-group differences in performance accuracy were not substantive. This is likely to reflect a speed/accuracy trade-off, which is common in self-paced tasks as patients are reluctant to make mistakes [5]. Thus while patients with CFS were able to perform at a comparable level of accuracy to healthy control participants, this was achieved at the expense of response speed. The subjective ratings of greater effort required to perform these tasks also confirmed that performance accuracy came at a cost for these patients.

A number of recent studies have reported associations between changes to white matter integrity and information processing speed [26]–[28]. While solid evidence supporting abnormalities in the structural integrity of white matter tracts in CFS is lacking, it could be argued that the prolonged response speed observed in our patients may reflect a loss in the capacity of white matter tracts to support efficient transmission of neural signals. However, if a loss in white matter integrity were responsible for our findings, one would expect to see a consistent reduction in response speed throughout a given test. Our data instead reveal significant variability in the speed of responses within each test. This is consistent with our previous findings of significant fluctuation in sustained attention and concentration in CFS [5] and point to an ongoing struggle to keep attention focused on the task at hand. Impairment in maintaining attention and concentration, as well as subtle deficits in executive functioning provides a plausible explanation of the current results and also fits with the reported cost of completing the tasks.

Our data do not support a relationship between overall fatigue levels and cognitive impairment. This is consistent with many studies in which subjective assessments of mental fatigue and cognitive complaints typically do not reflect their objective performance in cognitive tests [3], [5], [29]; although there are some exceptions [4]. As expected, patients with CFS reported higher levels of mental and physical fatigue and lower physical and mental energy compared to healthy control participants at both the baseline and post-testing assessment. Yet these ratings of energy and fatigue levels did not differentially change in response to task performance nor did they predict task performance. Our findings did also not support a role for psychological distress (symptoms of depression and anxiety) in cognitive dysfunction in CFS, which is consistent with several recent reports in the literature [6], [7].

The current data revealed a highly significant association between baseline HRV and performance outcomes - including response speed and objective performance indices within all tests. This novel finding adds to the body of evidence linking reduced HRV to the symptomatology in CFS. This finding is also consistent with previous reports demonstrating this association in healthy populations and community elders [15]–[17], [30]. Multiple regression analysis employed in this study identified baseline HRV as the only significant predictor of cognitive performance. As disease status was not an independent predictor, this suggests a broader role for low HRV in mental flexibility in general [15]–[17], [30].

### Dynamic HR Responsivity is Altered in Response to Cognitive Challenges in CFS

The substantively higher HR of patients with CFS seen both at rest and throughout the cognitive testing indicates that CFS is associated with a prevailing state of sympathetic hyper-arousal with concomitant response inflexibility [12]–[14]. More specifically, the between-group differences in the trajectory of HR responsivity not only supported the notion of sympathetic hyper-activity, but also a loss of sensitivity to task difficulty in patients with CFS.

Analysis of HRV data confirmed evidence for an overall reduction in cardiac vagal activity in patients with CFS [12]–[14]. With the onset of the cognitive stressor, control participants showed a more immediate adjustment in HRV, which then remained stable for the remainder of the testing session. In contrast, the HRV response to the stressor of patients with CFS was sluggish with a subsequent continuous, gradual decline in HRV throughout the session. Although reduced vagal activity during cognitive challenges has been described in healthy individuals [31], this is the first study indicating a differential vagal response in CFS compared with healthy control subjects in response to a series of cognitive challenges. The differences in HR and HRV are not likely to be a product of differing respiratory influences over the ANS expressed as sinus respiratory arrhythmia (the chief influence on HRV) as there were no between-group differences in the mean respiratory rates.

Patients with CFS also took significantly longer to recover to the baseline HR. The important role of the vagus in controlling low level cardiac adjustments in response to mental and physical stressors is well-described in the literature: withdrawal of vagal input (i.e. reduction in HRV) results in an increase in HR during stressful tasks and at the completion of the task, vagal input to the heart returns and rapidly restores HR to resting values [9], [10]. We speculate that while the dynamic vagal response in control participants allows them to regain resting HR quickly, the more sluggish vagal response seen in patients with CFS results in the delayed recovery time. This is the first reported evidence of a difference in time-to-recovery to resting HR after exposure to a mental stressor in CFS. This novel finding supports our results obtained from HRV and indicates that CFS is associated with a significant loss of vagal modulation which becomes particularly apparent when dealing with challenging tasks.

### Models of Neurovisceral Integration and CFS

The current results are consistent with the notion that CFS represents a ‘system under stress’ [2]. Several models of neurovisceral integration with specific reference to self-regulation, health and resilience have been articulated from different research contexts. Some of these models take a top-down perspective [30], [32], [33] emphasizing the influence of activity in higher cortical structures in response to challenges on downstream stress-responsive neural and physiological (including autonomic) systems. The prefrontal cortex (PFC) has long been accorded a pivotal role in integrating information from internal and external sources, synthesizing meaning for the individual, and initiating appropriate emotional and behavioural responses [34]. The PFC has been shown to exert important inhibitory control over limbic and physiological stress response systems [30], [32], [33]. PFC activity can be compromised by severe and/or persistent stress and trauma [30], [32] or by an encounter with potent uncontrollable stressors [32]. Loss of inhibitory control by the PFC has been linked to autonomic imbalance characterized by parasympathetic (vagal) loss, which can be indexed by HRV parameters [30], exaggerated emotional responsiveness [32], [35], and deficits in cognitive function particularly in attention, working memory and mental flexibility [30], [35].

Other neurovisceral integration models have focused on establishing the existence of afferent (bottom-up) pathways conveying signals from essentially all physiological systems and microenvironments (including inflammatory, metabolic, hormonal) first to autonomic and homeostatic centres, and then to higher limbic and cortical regions (including the anterior cingulate cortex, the insular and PFC). This dynamic stream of information endows the brain with a conscious awareness of the physiological condition of the entire body, termed interoception. It is postulated that in response to perceived homeostatic imbalances the brain adjusts emotions, motivated behaviours, and descending autonomic responses in order to maintain body integrity [34]–[37].

**Figure 4:**
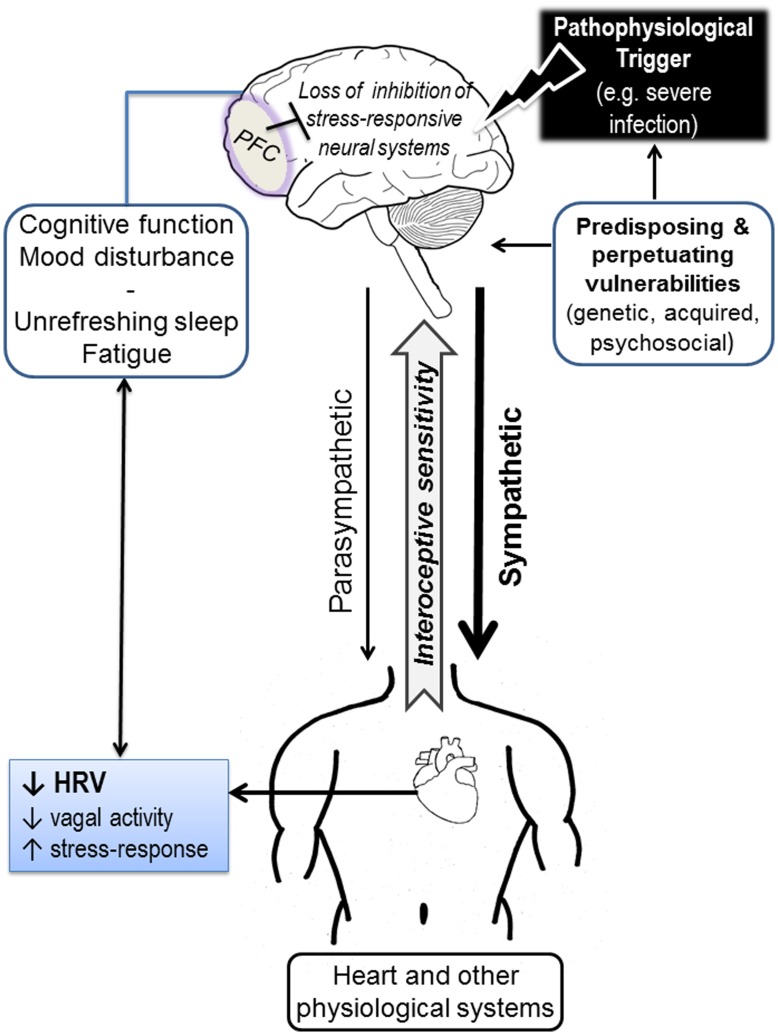
A hypothetical model of altered neurovisceral communication underlying the documented impairments in autonomic activation and behavioural self-regulation in CFS. PFC  =  prefrontal cortex; HRV  =  heat rate variability; CFS  =  chronic fatigue syndrome.

By combining elements of these models a speculative conceptualization of autonomic imbalance and its relationship to core symptoms in CFS may be constructed; this is shown in Figure 4. Although the aetiology of CFS is unknown, it is well-documented that a severe viral infection may trigger the condition in some individual [38]. We have therefore depicted ‘severe infection’ as a plausible pathophysiological trigger in the diagram. Existing vulnerabilities including genetic make-up, acquired/developmental sensitization in stress-response systems, personality, and psychosocial stressors are likely to interact with such a trigger to potentiate the severity of the acute stressor (e.g. the acute illness) [39] and thus the reduction of inhibitory control by the PFC. Vulnerable individuals may also perceive a severe illness as an uncontrollable stressor, which would further reduce PFC activity [32]. Loss of inhibitory control over stress-responsive neural structures is shown to result in a perturbation of autonomic outflow characterized by reduced vagal tone (reflected in low HRV) and heightened stress reactivity. Disturbance in physiological systems relating to the initial illness and/or the resulting imbalance in autonomic signalling is relayed to the brain via interoceptive pathways thus providing feedback, which may further perpetuate the loss of inhibitory control by the PFC resulting in an energy-costly state of physiological hyper-vigilance and stress reactivity (i.e. a system under stress). Behaviourally, such a state interferes with optimal self-regulation by a shift toward inflexible, pre-potent and, in the longer term, maladaptive response patterns to challenges. Although not part of the current findings we have previously shown heightened interoceptive sensitivity in patients with CFS [12] and have repeatedly identified low HRV as an important biological correlate of poor sleep quality in this patient group [14], [40].

Viewed within this framework, CFS can be conceptualized as a variant of post-traumatic stress disorder (PTSD), where the initial trigger is a severe internal stressor rather than a psychosocial trauma [12]. By analogy with PTSD, a clinical approach in CFS may seek to reconstitute the integrity of the neural circuits involved in the autonomic imbalance and associated disturbances in behavioural self-regulation. This may be achieved by various methods aimed to increase HRV and thus vagal tone [41]–[43]. Alternatively, transcranial direct current stimulation, which is currently optimized as a treatment for patients with depression (who also have documented low PFC activity) may hold potential as a technique for increasing activity in the PFC and improving cognitive difficulties [44].

### Limitations and Future Directions

The patients with CFS in this study were recruited from a treatment clinic providing an outpatient exercise pacing and cognitive therapy program. It is possible that these patients had higher levels of functioning than others not involved in such a program. This study found a significant association between reduced cardiac vagal activity and cognitive performance impairment in CFS. Nevertheless, exposition of the cause-effect relationship between these two variables, as well as of a critical role for inactivity of the PFC in the pathophysiology of CFS remains beyond the scope of this study and requires longitudinal cohort study designs incorporating neural imaging and/or electroencephalogram (EEG) studies. The current results were affected by the self-pacing format of our cognitive tests. While self-paced tests provide more accurate performance measures this is offset by a reduction in their utility as a stressor as subjects slow down, typically favouring accuracy over speed. Consequently, between-group differences in cardiac responsivity were not as pronounced as in a previous study by our group using a fixed-interval (1.5 sec) presentation of successive trials [12]. To optimally determine differences in cardiac responses to cognitive challenges in CFS, future studies need to devise tests that optimize their impact as a stressor while still permitting collection of accurate performance data.

It might be postulated that a specific increase in sympathetic drive is responsible for the increased HR during exposure to the stressor as patients with CFS showed a significant increase in HR in the absence of a dynamic reduction in HRV. By contrast, the findings in healthy participants in our study are consistent with previous reports in healthy individuals of a withdrawal of vagal input (i.e. reduction in HRV) and a concomitant increase in HR during stressful tasks [45]. However, as the current study did not employ direct assessment of cardiac sympathetic drive, increased cardiac sympathetic activity during exposure to stressors in CFS remains speculative.

### Conclusion

The findings in this study reveal for the first time a significant association between reduced cardiac vagal tone and cognitive impairment in CFS. This together with our recent report linking reduced HRV to the cardinal symptom of unrefreshing sleep and sleep problems in CFS provides further support for the role of autonomic disturbances in the symptomatology of CFS. A better understanding of the specific role of changes in autonomic functioning in the development and maintenance of symptoms in CFS awaits assessment in longitudinal cohort studies.
